# Phosphoregulation of the Titin-cap Protein Telethonin in Cardiac Myocytes*[Fn FN2]

**DOI:** 10.1074/jbc.M113.479030

**Published:** 2013-11-26

**Authors:** Alexandra J. Candasamy, Robert S. Haworth, Friederike Cuello, Michael Ibrahim, Sriram Aravamudhan, Marcus Krüger, Mark R. Holt, Cesare M. N. Terracciano, Manuel Mayr, Mathias Gautel, Metin Avkiran

**Affiliations:** From the ‡ Cardiovascular Division, King's College London British Heart Foundation Centre, London SE1 7EH, United Kingdom,; the § National Heart and Lung Institute, Imperial College, London W12 0NN, United Kingdom, and; the ¶ Max Planck Institute for Heart and Lung Research, 61231 Bad Nauheim, Germany

**Keywords:** CaMKII, Cardiac Muscle, Cardiomyopathy, Excitation-Contraction Coupling, Protein Kinase D (PKD), Protein Phosphorylation

## Abstract

Telethonin (also known as titin-cap or t-cap) is a muscle-specific protein whose mutation is associated with cardiac and skeletal myopathies through unknown mechanisms. Our previous work identified cardiac telethonin as an interaction partner for the protein kinase D catalytic domain. In this study, kinase assays used in conjunction with MS and site-directed mutagenesis confirmed telethonin as a substrate for protein kinase D and Ca^2+^/calmodulin-dependent kinase II *in vitro* and identified Ser-157 and Ser-161 as the phosphorylation sites. Phosphate affinity electrophoresis and MS revealed endogenous telethonin to exist in a constitutively bis-phosphorylated form in isolated adult rat ventricular myocytes and in mouse and rat ventricular myocardium. Following heterologous expression in myocytes by adenoviral gene transfer, wild-type telethonin became bis-phosphorylated, whereas S157A/S161A telethonin remained non-phosphorylated. Nevertheless, both proteins localized predominantly to the sarcomeric Z-disc, where they partially replaced endogenous telethonin. Such partial replacement with S157A/S161A telethonin disrupted transverse tubule organization and prolonged the time to peak of the intracellular Ca^2+^ transient and increased its variance. These data reveal, for the first time, that cardiac telethonin is constitutively bis-phosphorylated and suggest that such phosphorylation is critical for normal telethonin function, which may include maintenance of transverse tubule organization and intracellular Ca^2+^ transients.

## Teaser

**Background:** Telethonin mutations are associated with cardiomyopathy through unknown mechanisms.

**Results:** Telethonin is a substrate for CaMK family kinases and exists in a bis-phosphorylated state in cardiomyocytes, in which non-phosphorylated telethonin disrupts transverse tubule organization and intracellular calcium transients.

**Conclusion:** Telethonin phosphorylation is critical for the maintenance of normal cardiomyocyte morphology and calcium handling.

**Significance:** Disruption of phospho-telethonin functions may contribute to pathogenesis in cardiomyopathy.

## Introduction

Telethonin, which is also known as titin-cap or t-cap, is a 19-kDa protein that is expressed almost exclusively in cardiac and skeletal muscle, with a single isoform that is encoded by the *TCAP* gene and high sequence homology across species (1, 2). The N-terminal region of telethonin forms a unique β sheet structure in complex with the N-terminal Z1Z2 immunoglobulin-like domains of two titin molecules in a palindromic assembly, thus anchoring titin in the sarcomeric Z-disc (3, 4). The C-terminal region (or “tail”) appears unstructured even in complex with titin (5). A functional role for telethonin has been implicated in sarcomere development and stability (2, 6, 7), and mutations in *TCAP* are causally associated with both skeletal (8) and cardiac (9) myopathies. It has also been proposed that telethonin is involved in stretch sensing within the cardiac sarcomere (10) and that it may protect against cardiomyocyte apoptosis in hearts subjected to biomechanical stress (11). However, targeted deletion of *TCAP* in mice produces surprisingly subtle cardiac (11) and skeletal (12) phenotypes, suggesting that mechanisms more complex than loss of telethonin protein may contribute to genetic telethonin myopathies.

Little is known about the posttranslational mechanisms that may regulate telethonin function. Nevertheless, telethonin has been shown to be an *in vitro* substrate for the kinase domain of titin (titin kinase), an atypical member of the Ca^2+^/calmodulin-dependent kinase (CaMK)^5^ family that is, in fact, not activated by Ca^2+^/calmodulin (13) and that phosphorylates telethonin at a single C-terminal residue, Ser-157 (6). Furthermore, in a yeast two-hybrid screen of a human cardiac cDNA library, we have previously identified telethonin as an interaction partner and potential substrate for the catalytic domain of protein kinase D (PKD) (14), another atypical member of the CaMK family (15). In this study, we report that telethonin is indeed a substrate for PKD and also for CaMKII *in vitro*, map the phosphorylation sites to Ser-157 and a novel C-terminal phospho-acceptor at Ser-161, and show, for the first time, that endogenous telethonin is constitutively bis-phosphorylated in rat and mouse myocardium. Furthermore, through partial replacement of endogenous telethonin with a non-phosphorylatable mutant, we provide evidence that telethonin phosphorylation may regulate transverse tubule (t-tubule) organization and the intracellular Ca^2+^ (Ca^2+^*_i_*) transient in isolated ventricular myocytes. These findings shed new light on the potential functions and regulation of cardiac telethonin.

## EXPERIMENTAL PROCEDURES

Detailed methodology is provided in the supplemental material. Methods published previously were used for key techniques such as *in vitro* kinase assays (14), electron transfer dissociation tandem mass spectroscopy (16), the isolation and culture of ventricular myocytes from the adult rat heart (17), adenoviral vector construction and myocyte infection (18), immunoblot analysis (19), immunocytochemistry and fluorescence confocal microscopy (16, 20), and imaging and analysis of t-tubule structure and Ca^2+^*_i_* transients (21). Phosphate affinity SDS-PAGE utilized polyacrylamide-bound Mn^2+^-Phos-tag reagent (22, 23). Quantitative data are given as mean ± S.E., and intergroup comparisons were done by analysis of variance followed by the Newman-Keuls test. *p* < 0.05 was considered significant.

## RESULTS

**FIGURE 1.**
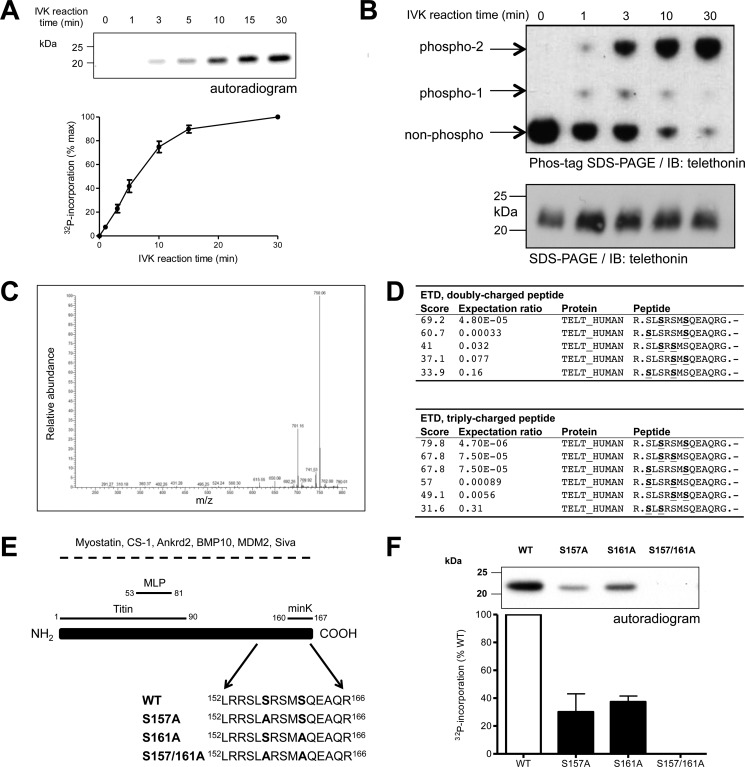
**PKD phosphorylates telethonin at Ser-157 and Ser-161.** *A*, time course of PKD-mediated phosphorylation of WT telethonin. Recombinant WT telethonin was incubated with PKD_cat_ and [^32^P]ATP and ^32^P incorporation over time was monitored by SDS-PAGE and autoradiography. *Top panel*, representative autoradiogram with quantitative data below (*n* = 4, mean ± S.E.). *IVK*, *in vitro* kinase. *B*, Phos-tag phosphate-affinity SDS-PAGE and immunoblot analysis of PKD-mediated phosphorylation of WT telethonin showing non-phosphorylated and phosphorylated moieties (*top panel*). Standard SDS-PAGE and immunoblot (*IB*) analysis of the same samples is also shown (*bottom panel*) (*n* = 3). *C*, collision-induced dissociation spectrum from the doubly charged, doubly phosphorylated telethonin fragment peptide showing the characteristic neutral losses (-49 and −98 Da) of two phosphate groups from the peptide. *D*, Mascot search results obtained for electron transfer dissociation (*ETD*) fragmentation spectra of the doubly and triply charged precursor. *S*, phosphorylated serine residue. *E*, schematic of telethonin, illustrating protein interactions reported previously. For interactions underscored by a *dashed line*, the telethonin domains involved have not been mapped. *Ankrd2*, ankyrin repeat domain-containing protein 2; *BMP10*, bone morphogenetic protein 10; *CS-1*, calsarcin 1; *MDM2*, murine double minute 2; *MLP*, muscle LIM protein. Also shown are amino acids 152–166 for all full-length recombinant telethonin proteins generated. *F*, representative autoradiogram showing PKD-mediated phosphorylation of recombinant WT and mutated telethonin proteins (*top panel*) with quantitative data below (*n* = 3, mean ± S.E.).

To verify our earlier work that suggested telethonin as a putative PKD substrate, recombinant human WT telethonin carrying an N-terminal His_6_ tag was used in an *in vitro* kinase assay with [γ-^32^P]ATP and PKD_cat_, a constitutively active form of the enzyme lacking the N-terminal regulatory domain (14). ^32^P was incorporated into telethonin in a time-dependent manner (Fig. 1*A*), confirming telethonin as an *in vitro* substrate for PKD. To explore the phospho-telethonin species generated, we also analyzed PKD-mediated phosphorylation by Phos-tag phosphate affinity SDS-PAGE in combination with immunoblot analysis (22, 23). As the duration of the phosphorylation reaction increased, WT telethonin was found to transition almost completely from the non-phosphorylated form to a slow-migrating, fully phosphorylated form, with an intermediate phospho-telethonin moiety also appearing transiently during the first 10 min of the reaction and suggesting the existence of multiple PKD phosphorylation sites (Fig. 1*B*). To identify the phospho-acceptor residues that are targeted by PKD, recombinant human WT telethonin was subjected to trypsin digestion following a 30-min phosphorylation *in vitro* by PKD_cat_. Analysis by nanoflow-liquid chromatography-tandem mass spectrometry revealed two phosphorylated residues, with characteristic neutral losses (-49 and −98 Da) observed upon fragmentation by collision-induced dissociation (Fig. 1*C*). The pertinent peptide fragment was further analyzed by electron transfer dissociation, and the serine residues Ser-157 and Ser-161 were identified as the putative phospho-acceptor sites (Fig. 1*D*). To confirm that Ser-157 and Ser-161 are indeed targeted by PKD, further *in vitro* kinase assays were performed, using as a substrate either WT telethonin protein (as above) or a mutated telethonin with replacement of the putative phospho-acceptor serine residues by non-phosphorylatable alanine, either individually or in combination (S157A, S161A, or S157A/S161A; partial sequences are shown in Fig. 1*E*). Although WT telethonin protein was robustly phosphorylated by PKD, as before, the presence of each single Ser/Ala substitution (S157A or S161A) partially attenuated such phosphorylation (Fig. 1*F*). Furthermore, PKD-mediated phosphorylation was completely abolished by the double mutation (S157A/S161A) (Fig. 1*F*). These findings show that telethonin is phosphorylated at both Ser-157 and Ser-161 by PKD *in vitro* and that no other potential phospho-acceptor residues in telethonin are targeted under these conditions. This was confirmed by complementary Phos-tag phosphate affinity SDS-PAGE and immunoblot analysis. When non-phosphorylated, WT and mutated telethonin proteins migrated as a single band of identical mobility in a Phos-tag SDS-PAGE gel (supplemental Fig. S1). Following PKD-mediated phosphorylation of WT, S157A, or S161A telethonin, an additional, slower-migrating protein band became apparent and was the predominant species in each case (supplemental Fig. S1). Importantly, the in-gel mobility of the phospho-telethonin species varied between WT, S157A, and S161A telethonin (supplemental Fig. S1), reflecting the differences in the number and position of the phosphorylated serine residue(s). Furthermore, S157A/S161A mutant telethonin, when exposed to PKD and ATP like WT telethonin, migrated as a single band with an in-gel mobility identical to that of non-phosphorylated S157A/S161A or WT telethonin (supplemental Fig. S1), thus confirming the absence of additional PKD target sites other than Ser-157 and Ser-161.

**FIGURE 2.**
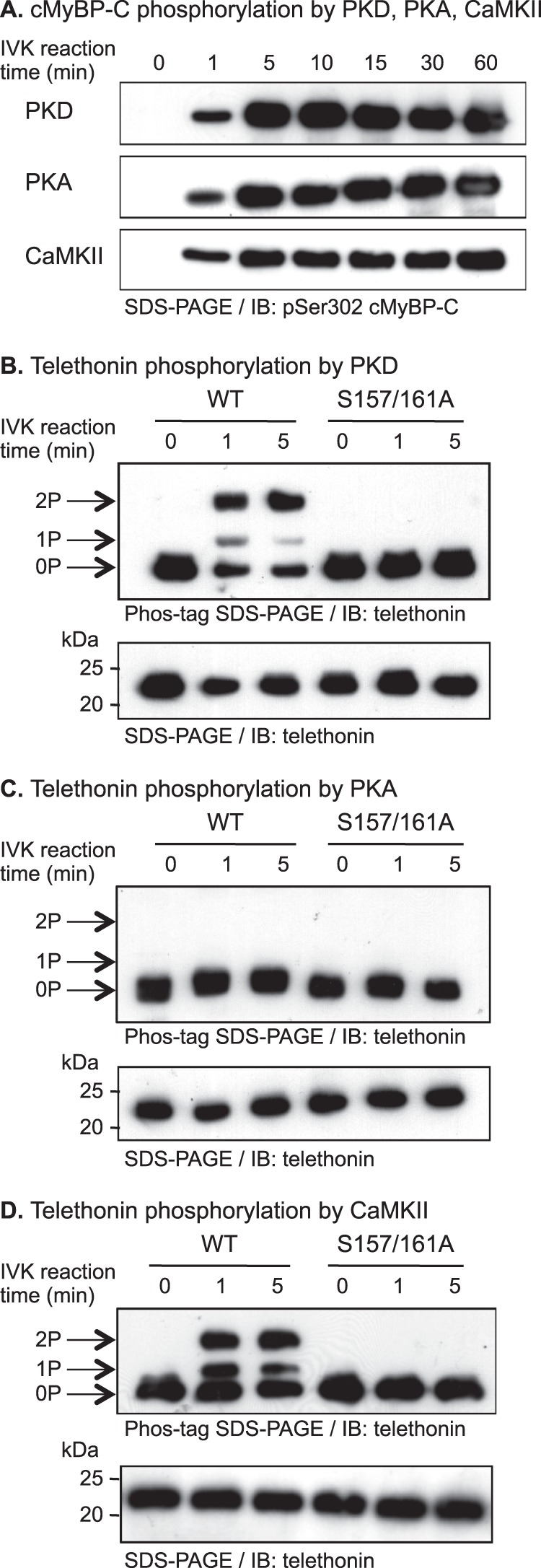
**Telethonin is phosphorylated at Ser-157 and Ser-161 by PKD and CaMKII but not by PKA.** *A*, comparable phosphorylation by PKD, PKA, or CaMKII of Ser-302 in the recombinant cardiac myosin-binding protein C (*cMyBP-C*) c1c2 fragment *in vitro*. A recombinant His_6_-tagged c1c2 fragment (100 pmol) was incubated in the presence of each kinase for 0–60 min and subjected to immunoblot (*IB*) analysis using a phospho-specific pSer-302 cMyBP-C antibody. *IVK*, *in vitro* kinase. *B–D*, differential phosphorylation by PKD (*B*), PKA (*C*), or CaMKII (*D*) of recombinant telethonin *in vitro*. Recombinant His_6_-tagged WT or S157A/S161A telethonin proteins (100 pmol) were incubated in the absence or presence of each kinase for 0, 1, or 5 min and subjected to Phos-tag SDS-PAGE and immunoblot analysis using a monoclonal anti-telethonin antibody (*top panel*). *Arrows* on the *left* show the identities of dual-phosphorylated (*2P*), mono-phosphorylated (*1P*), and non-phosphorylated (*0P*) telethonin. The *bottom panel* shows the same samples subjected to standard SDS-PAGE and immunoblot analysis using the same antibody.

We next examined the *in vitro* phosphorylation of telethonin by PKD *versus* Ca^2+^/calmodulin-dependent kinase II (CaMKII, α isoform) and PKA. In conditions under which PKD, CaMKII, and PKA catalyzed comparable phosphorylation of an established common substrate, Ser-302 of cardiac myosin-binding protein C (cMyBP-C) (20, 24), PKD and CaMKII induced similar phosphorylation of telethonin, indicating that both kinases target Ser-157 and Ser-161, whereas PKA was without effect (Fig. 2). It appears, therefore, that telethonin may be a target for multiple members of the CaMK family to which titin kinase, PKD, and CaMKII all belong (but PKA does not).

**FIGURE 3.**
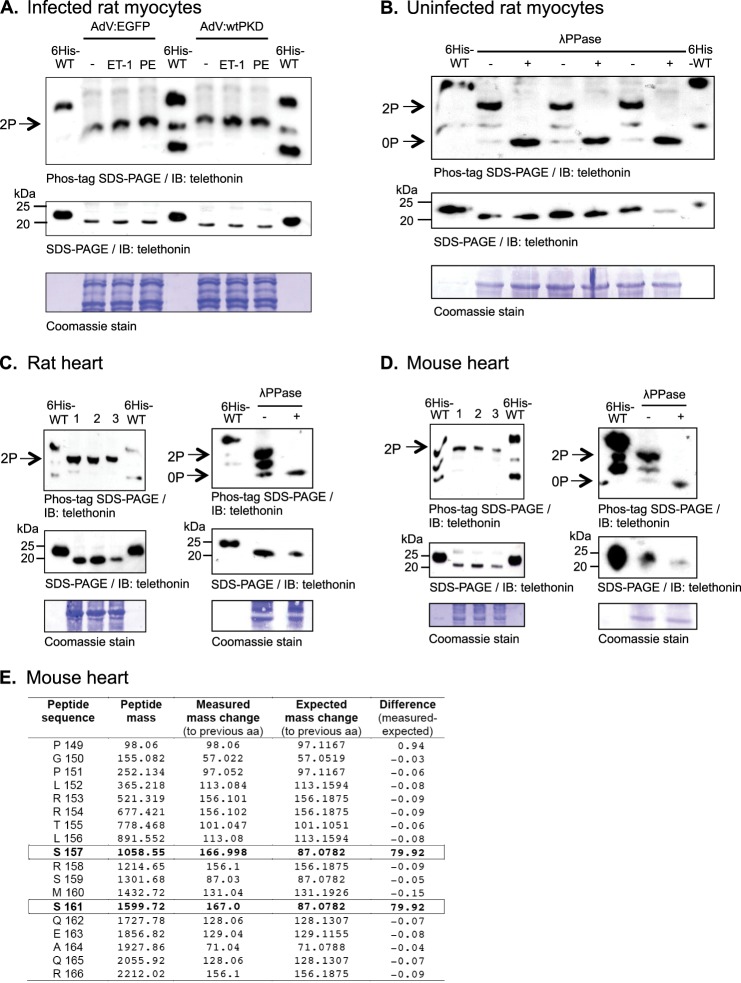
**Telethonin is constitutively phosphorylated in ventricular myocytes and tissue.** *A*, Phos-tag SDS-PAGE and immunoblot analysis using the monoclonal anti-telethonin antibody of samples from ARVM infected with AdV:EGFP or AdV:wtPKD and stimulated with 100 nm ET-1 or 10 μm PE (with 1 μm atenolol) for 10 min. The same samples were subjected to standard SDS-PAGE and immunoblot (*IB*) analysis using the same antibody, and membranes were stained after use with Coomassie, as indicated. *2P*, dual-phosphorylated. *B*, Phos-tag SDS-PAGE and immunoblot analysis using the monoclonal anti-telethonin antibody of samples from uninfected ARVM incubated (30 min at 30 °C) in the absence (-) or presence (+) of λPPase. The same samples were subjected to standard SDS-PAGE and immunoblot analysis using the same antibody, and membranes were stained after use with Coomassie as indicated. *0P*, non-phosphorylated. *C* and *D*, Phos-tag SDS-PAGE and immunoblot analysis using the monoclonal anti-telethonin antibody of homogenates from rat (*C*) or mouse (*D*) ventricular tissues from three separate hearts in each case (*left panels*). Data are also shown for rat (*C*) and mouse (*D*) ventricular samples incubated (30 min at 30 °C) in the absence (-) or presence (+) of λPPase (*right panels*). The same samples were subjected to standard SDS-PAGE and immunoblot analysis using the same antibody, and membranes were stained after use with Coomassie as indicated. In *A–D*, recombinant His_6_-tagged WT telethonin (100 pmol) phosphorylated *in vitro* by PKD_cat_ for 3 min was included as an internal standard in the gels. *E*, summary data from Fourier transform high-energy collisional dissociation MS sequencing of a bis-phosphorylated telethonin phosphopeptide fragment from mouse myocardium (all masses in Dalton). A mass difference from the expected mass of ∼80 Da (equivalent to -PO_3_) is observed at both Ser-157 and Ser-161, indicating phosphorylation at these residues. *aa*, amino acid.

The phosphorylation status of endogenous telethonin in isolated adult rat ventricular myocytes (ARVM) was then investigated in the absence or presence of increased cellular PKD activity. Increased cellular PKD activity was achieved by overexpression of WT PKD1 using an adenoviral vector (AdV:wtPKD) (18) and its activation by cellular stimulation with endothelin 1 (ET-1) or phenylephrine (PE). Preliminary experiments showed that ARVM infected with AdV:wtPKD showed increased PKD expression (relative to control cells infected with AdV:EGFP) and that both ET-1 and PE markedly activated heterologously expressed PKD, as reflected by its increased phosphorylation at Ser-744/748 and Ser-916 (supplemental Fig. S2). Phos-tag SDS-PAGE and immunoblot analysis of the same samples showed that, in all treatment groups, endogenous telethonin migrated as a single species whose in-gel mobility (and, therefore, phosphorylation status) was unaffected by increased PKD expression and activity (Fig. 3*A*, *top panel*). By reference to His_6_-tagged WT telethonin included in the same gel as an internal standard, this endogenous telethonin species migrated between bis-phosphorylated (pSer-157/161) and mono-phosphorylated (pSer-161) recombinant telethonin moieties (Fig. 3*A*, *top panel*). Importantly, standard SDS-PAGE and immunoblot analysis of the same samples revealed that endogenous telethonin migrates more rapidly than the recombinant internal standard (Fig. 3*A*, *center panel*), most likely because of the presence of additional N-terminal residues in the latter. This led us to conclude that the endogenous telethonin species that migrates between the positions of the mono-phosphorylated and bis-phosphorylated recombinant internal standards in Phos-tag SDS-PAGE gels (Fig. 3*A*, *top panel*) is likely to represent the bis-phosphorylated form of the protein. To confirm the identity of this predominant telethonin moiety, we also performed Phos-tag SDS-PAGE analysis of protein samples extracted from uninfected ARVM in the absence of phosphatase inhibitors with and without subsequent *in vitro* incubation with λ phosphatase (λPPase). In the absence of λPPase treatment, the predominant telethonin moiety once again appeared to be bis-phosphorylated, although additional species likely to represent mono-phosphorylated and non-phosphorylated telethonin were also detectable (Fig. 3*B*, *top panel*). Importantly, following λPPase treatment, only a fast-migrating species representing non-phosphorylated telethonin was detectable (Fig. 3*B*, *top panel*). From this, we concluded that endogenous telethonin in isolated ARVM is bis-phosphorylated constitutively so that its phosphorylation status cannot be further enhanced by increased cellular PKD activity.

To further explore the phosphorylation status of endogenous telethonin, this was also investigated in ventricular tissue from rat (Fig. 3*C*) and mouse (*D*) hearts. In these samples, endogenous telethonin also displayed a similar migration profile (relative to the recombinant internal standard) to that observed in isolated ARVM maintained in culture. Furthermore, λPPase treatment caused a comparable shift in the migration profile of the predominant species. These observations suggest that endogenous telethonin exists primarily in a constitutively bis-phosphorylated form in isolated ARVM and in the intact rat or mouse heart. To confirm the identity of the phosphorylated residues in endogenous telethonin, phosphoprotein fragments from mouse myocardium were additionally subjected to Fourier transform MS analysis with high-energy collisional dissociation (supplemental Fig. S3). This confirmed that endogenous telethonin is constitutively phosphorylated at both Ser-157 and Ser-161 in mouse myocardium *in vivo* (Fig. 3*E*).

To facilitate investigation of the functional importance of telethonin phosphorylation, adenoviral vectors were constructed to allow expression of HA-tagged WT or non-phosphorylatable telethonin (AdV:HA-WT-telethonin and AdV:HA-S157A/S161A-telethonin, respectively) in ARVM. SDS-PAGE and immunoblot analysis of lysates from ARVM infected with each adenovirus at varying multiplicities of infection (0–1000 plague-forming units/cell) for 18 or 42 h allowed selective detection of heterologously expressed telethonin with an anti-HA antibody and simultaneous detection of both endogenous and heterologously expressed telethonin with an anti-telethonin antibody (because of slower migration of the HA-tagged protein). Heterologous telethonin was barely detectable 18 h post-infection (data not shown) but was robustly expressed 42 h post-infection (supplemental Fig. S4). A virus dose of 100 plaque-forming units/cell was selected for both AdV:HA-WT-telethonin and AdV:HA-S157A/S161A-telethonin for use in subsequent experiments because this produced similar levels of expression of each heterologous protein (supplemental Fig. S4, *top panel*), with an abundance roughly comparable with that of endogenous telethonin (supplemental Fig. S4, *center panel*).

**FIGURE 4.**
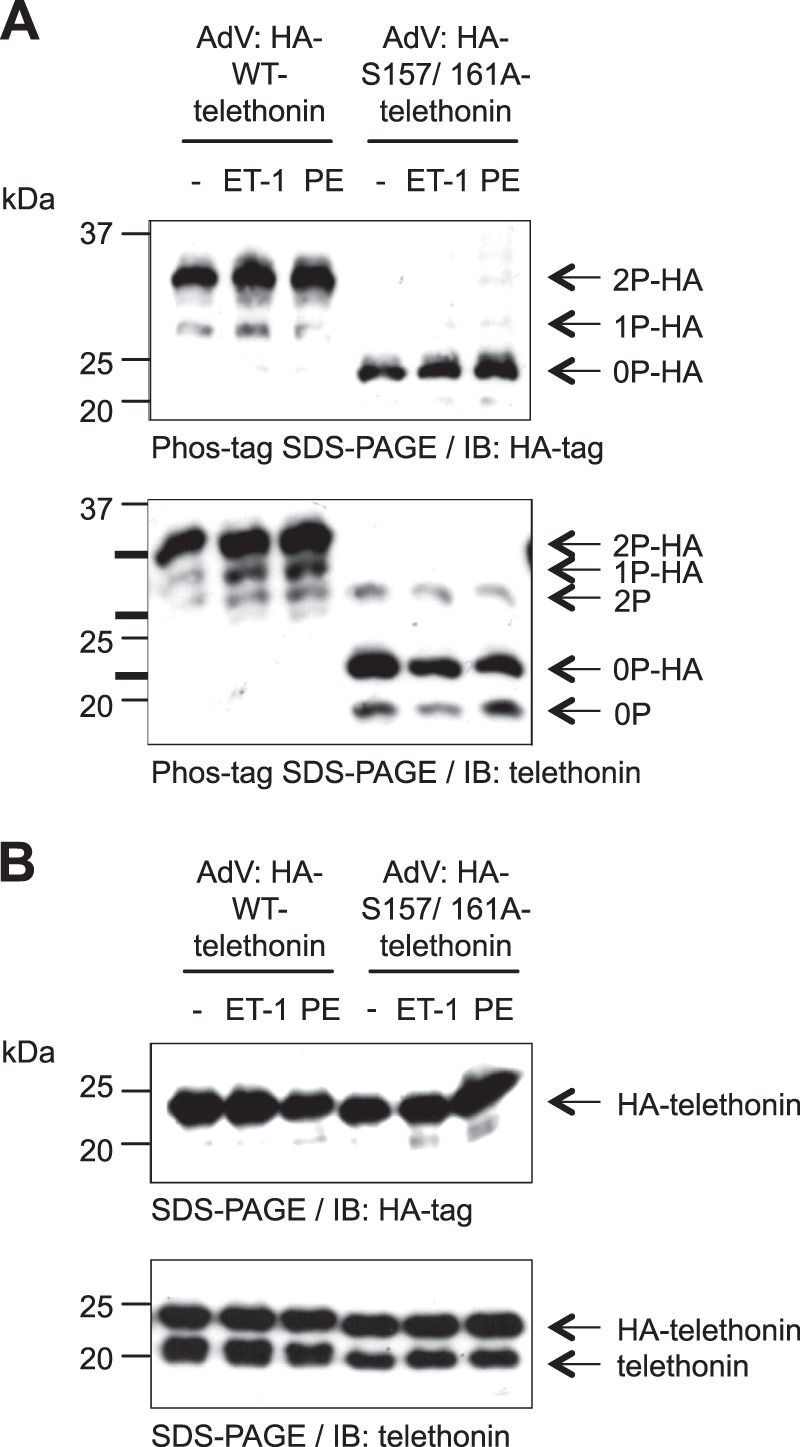
**Heterologously expressed WT telethonin and endogenous telethonin are constitutively phosphorylated.** *A*, Phos-tag SDS-PAGE and immunoblot (*IB*) analysis of ARVM infected with AdV:HA-WT-telethonin or AdV:HA-S157A/S161A-telethonin for 42 h and stimulated with vehicle, ET-1 (100 nm), or PE (10 μm, with 1 μm atenolol) for 10 min. Cell lysates were subjected to Phos-tag SDS-PAGE and immunoblot analysis using a monoclonal anti-HA antibody (*top panel*) or a monoclonal anti-telethonin antibody (*bottom panel*). The positions of internal standards resolved in the same gel are marked by *thick black lines* to the *left* of the immunoblot obtained with the anti-telethonin antibody, indicating the positions of dual-phosphorylated, mono-phosphorylated, and non-phosphorylated His_6_-tagged WT telethonin from *top* to *bottom. Arrows* on the *right* show the deduced identities of the indicated telethonin species. *2P-HA*, dual-phosphorylated HA-tagged heterologous telethonin; *1P-HA*, mono-phosphorylated HA-tagged heterologous telethonin; *0P-HA*, non-phosphorylated HA-tagged heterologous telethonin; *2P*, dual-phosphorylated endogenous telethonin; *0P*, non-phosphorylated endogenous telethonin. *B*, the same samples were subjected to standard SDS-PAGE and immunoblot analysis using the same antibodies (*n* = 3).

We first characterized the phosphorylation status of heterologously expressed, HA-tagged WT and S157A/S161A telethonin in ARVM in the absence or presence of ET-1 or PE. Phos-tag SDS-PAGE and immunoblot analysis with the anti-HA antibody revealed a single predominant band in ARVM expressing HA-WT-telethonin or HA-S157A/S161A-telethonin (Fig. 4*A*, *top panel*). Importantly, however, HA-WT-telethonin displayed markedly slower migration than HA-S157A/S161A-telethonin (Fig. 4*A*, *top panel*), indicating that it becomes phosphorylated following its cellular expression. With the anti-HA antibody, an additional, faster migrating moiety of lower abundance was also detected in cells expressing HA-WT-telethonin (Fig. 4*A*, *top panel*) that likely represents a mono-phosphorylated species. Nevertheless, no additional HA-tagged protein moiety became apparent in response to stimulation of ARVM expressing HA-WT-telethonin with ET-1 or PE (Fig. 4*A*, *top panel*), indicating that the available phospho-acceptor residues in heterologously expressed WT telethonin become fully phosphorylated by constitutive kinase activity. Additionally, HA-S157A/S161A-telethonin also remained as a single predominant band that displayed markedly faster migration relative to HA-WT-telethonin, regardless of stimulation with ET-1 or PE (Fig. 4*A*, *top panel*), suggesting that signaling pathways activated by these stimuli do not lead to phosphorylation of residues distinct from Ser-157 and Ser-161. These findings confirm that heterologously expressed WT telethonin, like endogenous telethonin, becomes constitutively bis-phosphorylated in ARVM, even in the absence of neurohormonal stimulation, and that such phosphorylation occurs at Ser-157 and Ser-161.

When Phos-tag SDS-PAGE was followed by immunoblot analysis with the anti-telethonin antibody (instead of the anti-HA antibody), protein species representing endogenous telethonin could also be visualized. Interestingly, although a constitutively bis-phosphorylated endogenous telethonin species was present in ARVM heterologously expressing either HA-WT-telethonin or HA-S157A/S161A-telethonin, a non-phosphorylated endogenous telethonin species became apparent only in the latter group (Fig. 4*A*, *bottom panel*). This observation suggests that the heterologous expression of non-phosphorylatable HA-S157A/S161A-telethonin may have an inhibitory effect on the phosphorylation of endogenous telethonin, likely by acting as a pseudosubstrate inhibitor. In these experiments, additional analysis of the same samples by standard SDS-PAGE and immunoblotting (with either the anti-HA or the anti-telethonin antibody) confirmed that HA-WT-telethonin and HA-S157A/S161A-telethonin were expressed at equivalent levels, each at an abundance that was roughly comparable with that of endogenous telethonin (Fig. 4*B*).

**FIGURE 5.**
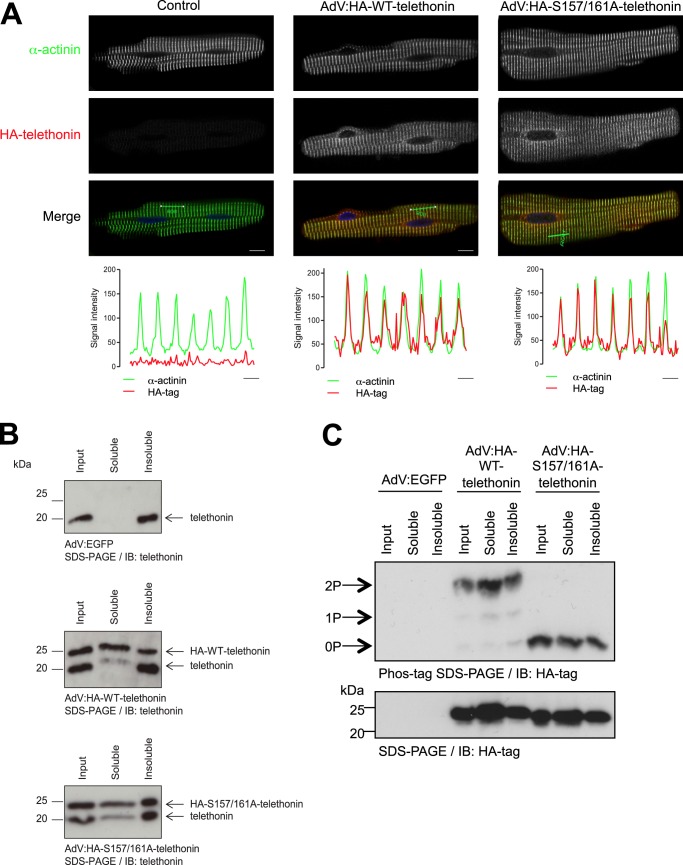
**HA-WT-telethonin and HA-Ser-157/161-telethonin localize to the sarcomeric Z-disc and partially displace endogenous telethonin.** *A*, confocal microscopy images of ARVM infected with AdV:HA-WT-telethonin or AdV:HA-S157A/S161A-telethonin for 42 h. Fixed and permeabilized cells were immunolabeled with rat monoclonal anti-HA and mouse monoclonal anti-α-actinin primary antibodies and Cy3-anti-rat and Cy5-anti-mouse secondary antibodies, and confocal microscopy was used to image infected myocytes. Cy3 (HA-telethonin) and Cy5 (α-actinin) images are shown in separate channels, with merged images showing α-actinin (*green*), HA-telethonin (*red*), and nuclei stained with DAPI (*blue*). The *line charts* show the intensity plots of the Cy3 (HA-telethonin) and Cy5 (α-actinin) signals over seven sarcomeres (across the regions shown by a *green line* on the *merged images*). *Scale bars* = 10 μm (confocal images) and 2 μm (intensity plots). *n* = 4, 5 cells/experiment. *B*, distribution of endogenous and heterologous telethonin species in Triton-soluble and insoluble fractions of ARVM infected with AdV:EGFP, AdV:HA-WT-telethonin, or AdV:HA-S157A/S161A-telethonin for 42 h. *IB*, immunoblot. *C*, phosphorylation of heterologous telethonin species in Triton-soluble and insoluble fractions of ARVM infected with AdV:EGFP, AdV:HA-WT-telethonin, or AdV:HA-S157A/S161A-telethonin for 42 h. *2P,* dual-phosphorylated; *1P,* mono-phosphorylated; *0P,* non-phosphorylated.

We then utilized ARVM in which heterologously expressed HA-WT-telethonin becomes constitutively bis-phosphorylated, whereas HA-S157A/S161A-telethonin cannot be phosphorylated (Fig. 4*A*), to explore the potential impact of phosphorylation on the subcellular localization and functions of telethonin. Immunolabeling with the HA tag antibody and confocal microscopy revealed that both HA-WT-telethonin and HA-S157A/S161A-telethonin were targeted predominantly to the cytoplasm and concentrated at the Z-disc, where they colocalized with the established Z-disc marker α-actinin (Fig. 5*A*). Within the spatial resolution of this technique, the subcellular localization of telethonin, therefore, does not appear to be regulated by its phosphorylation status. Endogenous telethonin is anchored to the detergent-insoluble myofilament compartment through superstable interactions of its N terminus with the N termini of two titin molecules at the sarcomeric Z-disc (3, 4, 25). To explore whether heterologously expressed telethonin is able to replace endogenous telethonin in this compartment, we performed detergent-based fractionation of ARVM proteins following heterologous expression of HA-WT-telethonin or HA-S157A/S161A-telethonin. As expected, in control cells (infected with AdV:EGFP), endogenous telethonin resided exclusively in the Triton-insoluble fraction (Fig. 5*B*, *top panel*). In contrast, in ARVM infected with AdV:HA-WT-telethonin (Fig. 5*B*, *center panel*) or AdV:HA-S157A/S161A-telethonin (*bottom panel*), endogenous telethonin was detected in both the Triton-insoluble and the Triton-soluble fractions. Furthermore, in these cells, HA-WT-telethonin or HA-S157A/S161A-telethonin was also found in both the Triton-insoluble and the Triton-soluble fractions (Fig. 5*B*), and the phosphorylation status of each protein was comparable in either fraction (*C*). These findings suggest that, when expressed at an abundance comparable with that of the endogenous protein, heterologous HA-tagged telethonin replaces a marked proportion of endogenous telethonin in the titin-telethonin complex and that such replacement occurs independently of the phosphorylation status of the heterologously expressed telethonin. Indeed, across multiple independent experiments, the heterologously expressed protein represented 58 ± 9% and 68 ± 8% of total telethonin in the Triton-insoluble fraction of cells infected with AdV:HA-WT-telethonin or AdV:HA-S157A/S161A-telethonin, respectively (*n* = 10, non-significant).

**FIGURE 6.**
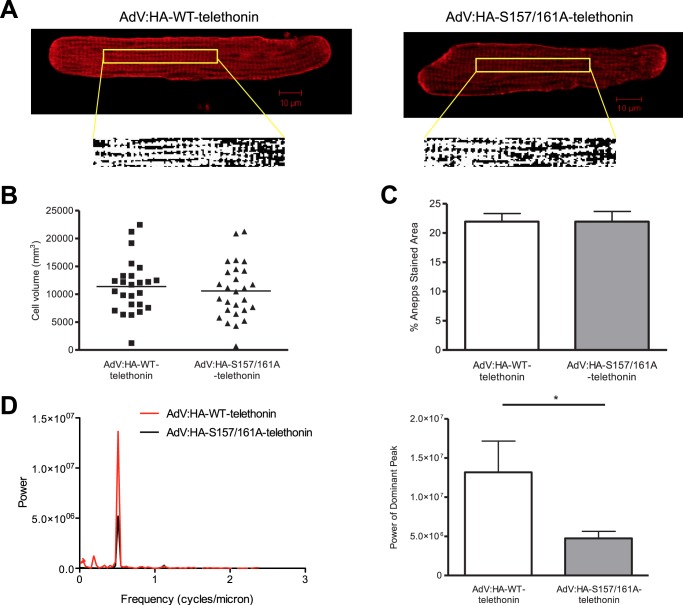
**Telethonin phosphorylation may regulate t-tubule organization.** *A*, representative confocal fluorescence images of ARVM infected with AdV:HA-WT-telethonin or AdV:HA-S157/161-telethonin for 42 h and labeled with di-8-ANEPPS. Binarized images of a central area, used for Fourier analysis of t-tubule regularity, are also shown. *B*, cell volume as assessed by Z-stack analysis of confocal fluorescence images. *C*, density of t-tubule network as reflected by the percentage of the cell interior labeled with di-8-ANEPPS. *D*, Fourier analysis of binarized di-8-ANEPPS images showing a representative power spectrum (*left panel*) and quantitative data (*right panel*). *, *p* < 0.05; *n* = 25–28 cells/group.

The C-terminal portion of telethonin, which contains the phosphorylation sites that we have identified, has been shown to interact with ion channel accessory proteins that reside in t-tubule membranes, leading to the suggestion that telethonin may serve as an “adapter protein” that links t-tubules to the Z-disc complex in cardiac myocytes (26, 27). Furthermore, telethonin knockdown has been reported to interfere with t-tubule development in striated muscle of zebrafish embryos (28), and, recently, telethonin knockout has been shown to disrupt t-tubule structure and function in mouse cardiomyocytes (29). Therefore, we took advantage of our ability to partially replace endogenous, phosphorylated telethonin with heterologous, non-phosphorylatable telethonin to explore the potential role of telethonin phosphorylation in regulating t-tubule structure and organization in ARVM. To this end, ARVM expressing HA-WT-telethonin or HA-S157A/S161A-telethonin were labeled with the membrane dye di-8-ANEPPS to visualize the t-tubule network (Fig. 6*A*). No differences in cell volume (Fig. 6*B*) or the density of the t-tubule network (*C*) were observed between the two groups. However, Fourier analysis of binarized di-8-ANEPPS-labeled images revealed significantly reduced power of the peak corresponding to the dominant frequency of t-tubule periodicity (used previously as an index of t-tubule regularity (30)) in cells expressing HA-S157A/S161A-telethonin relative to those expressing HA-WT-telethonin (Fig. 6*D*). This observation suggests that phosphorylation of telethonin at Ser-157 and Ser-161 may regulate the organization of the t-tubule network so that this is disrupted by reduced phosphorylation. Similar Fourier analysis of the distribution of α-actinin and myomesin, established markers of the Z-disc and the M-band, following immunolabeling confirmed the absence of general cellular disorganization and the maintenance of an identical average sarcomere length of ∼1.8 μm on the basis of the position of the dominant frequency (supplemental Fig. S5).

**FIGURE 7.**
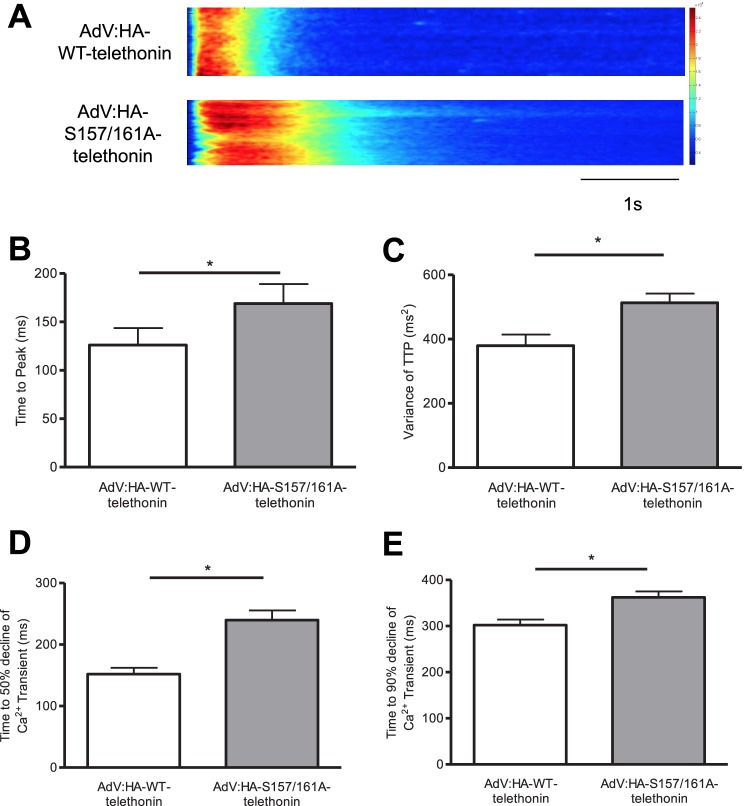
**Telethonin phosphorylation may regulate intracellular Ca^2+^ transients.** *A*, representative whole-cell Ca^2+^*_i_* transients measured in confocal line scan mode in ARVM infected with AdV:HA-WT-telethonin or AdV:HA-S157//161A-telethonin for 42 h. ARVM were loaded with the Ca^2+^-sensitive dye Fluo-4 and field-stimulated at 1 Hz. The quantitative data shown are the average time to peak of the Ca^2+^*_i_* transient (*B*), the variance of the time to peak (*C*), and the average time to 50% (*D*) and 90% (*E*) decline of the Ca^2+^*_i_* transient. *, *p* < 0.05; *n* = 25–28 cells/group.

The changes in t-tubule organization that arise from partial replacement in ARVM of endogenous, bis-phosphorylated telethonin with heterologous, non-phosphorylatable telethonin resemble those that have been reported to occur in rodent heart failure models (30, 31) in which t-tubule remodeling has been associated with abnormal Ca^2+^*_i_* transients in isolated ventricular myocytes (27). Therefore, we also explored the impact of partial replacement of endogenous, bis-phosphorylated telethonin with heterologous, non-phosphorylatable telethonin on the Ca^2+^*_i_* transient in ARVM. The dynamics and synchronicity of the whole-cell Ca^2+^*_i_* transient were altered in cells expressing HA-S157A/S161A-telethonin relative to those expressing HA-WT-telethonin (Fig. 7*A*). This was reflected by a prolonged average time to peak of the Ca^2+^*_i_* transient (Fig. 7*B*), despite an increase in amplitude (in units of peak/baseline fluorescence ratio from 1.45 ± 0.04 (*n* = 25) to 1.72 ± 0.07 (*n* = 28), *p* < 0.05), and an increased variance of time to peak of the Ca^2+^*_i_* transient (*C*). Additionally, the decay of the Ca^2+^*_i_* transient was slowed, as reflected by increased average times to 50% (Fig. 7*D*) and 90% (*E*) decline in cells expressing HA-S157A/S161A-telethonin. These findings suggest that the phosphorylation status of telethonin may affect the synchronous release and removal of Ca^2+^*_i_* in time and space during excitation-contraction coupling, likely as a consequence of altered t-tubule organization.

## DISCUSSION

The key novel observations that arise from this study are as follows. Ser-157 and Ser-161 in the C-terminal tail of telethonin are substrates for the CaMK family members PKD and CaMKII. These sites are maintained in a constitutively phosphorylated state in cardiac telethonin. A genetic intervention that partially replaces endogenous bis-phosphorylated telethonin in its native subcellular compartment with mutated non-phosphorylatable telethonin, but not with WT telethonin that itself becomes bis-phosphorylated, disrupts cardiomyocyte t-tubule organization and synchronous activation and dynamics of the Ca^2+^*_i_* transient. These findings provide new information on the regulation of cardiac telethonin and the potential functional roles of its C-terminal phosphorylation. They also raise a number of interesting questions.

Firstly, what are the mechanism(s) through which cardiac telethonin phosphorylation is regulated? Our studies with *in vitro* phosphorylation of recombinant telethonin definitively identify Ser-157 and Ser-161 as PKD substrates. Nevertheless, increased PKD activity in ARVM was not associated with increased telethonin phosphorylation because the endogenous protein existed predominantly in a constitutively bis-phosphorylated state, even under basal conditions. Furthermore, telethonin phosphorylation was not reduced by incubation of ARVM for up to 6 h with a bipyrydyl PKD inhibitor that exhibits remarkable efficacy and selectivity (22) (data not shown). Therefore, the contribution of PKD activity to the constitutive phosphorylation of cardiac telethonin remains unclear, particularly because other members of the CaMK family, such as titin kinase (6) and CaMKII (this study), can also catalyze telethonin phosphorylation at Ser-157 alone (6) or both Ser-157 and Ser-161, respectively. Regardless of the identity of the kinases that catalyze telethonin phosphorylation, our findings suggest that bis-phosphorylated telethonin has a slow turnover and may thus be relatively resistant to dephosphorylation. This could occur potentially because phosphorylation at Ser-157 and Ser-161 creates a binding site for a partner protein whose association with telethonin shields the pertinent phospho-acceptor residues from phosphatase action. To our knowledge, the only protein that has been reported previously to interact with the C-terminal tail of telethonin in a manner that is dependent on the phosphorylation status of the latter is the β subunit of the K^+^ channel that carries the slow component of the cardiac delayed rectifier current (26). This subunit, known as minK (also called KCNE1), was found to interact with telethonin in a yeast two-hybrid assay, with the interaction abolished by phosphomimetic S157E and S157D substitutions in telethonin (26). Nevertheless, even if a minK/telethonin interaction were to occur in cardiomyocytes (for which there is currently no direct evidence, especially given that minK expression is restricted to the conduction system (32)), the indication that this interaction is abrogated by telethonin phosphorylation at Ser-157 (26) makes it unlikely that association with minK underlies the apparent resistance of phospho-telethonin to phosphatase action. Archetypal proteins that bind to phosphorylated motifs on partner proteins are members of the 14-3-3 family, with several recent examples that 14-3-3 binding may protect binding site phosphorylated residues from dephosphorylation (33, 34). However, by using a “far-Western” overlay assay (35), we have found no evidence of 14-3-3 protein binding to WT or mutated (S157A, S161A, S157A/S161A) recombinant telethonin proteins in the absence or presence of PKD-mediated phosphorylation (data not shown). Thus, at present, the cellular mechanisms that are responsible for maintaining cardiac telethonin in a constitutively bis-phosphorylated state remain unknown. Interestingly, mono-phosphorylated and non-phosphorylated telethonin species became more readily detectable following the extraction and incubation of proteins from ARVM, rat heart, and mouse heart in the absence of phosphatase inhibitors (Fig. 3), suggesting that endogenous phosphatase(s) do indeed target phosphorylated Ser-157 and Ser-161.

Secondly, what is the physiological significance of telethonin being maintained in a constitutively bis-phosphorylated state? Our data with partial replacement of endogenous bis-phosphorylated telethonin with heterologous non-phosphorylatable telethonin in ARVM suggest that telethonin phosphorylation is important in maintaining t-tubule organization. A possible mechanism for such regulation of t-tubule organization is that telethonin indeed serves as an adapter protein that anchors t-tubules to Z-discs, as Furukawa *et al.* (26) have suggested, but that phosphorylation is necessary for telethonin to serve this function. To fulfil such an anchoring role, the phosphorylated C terminus of telethonin would have to interact with partner protein(s) localized to the t-tubule membrane. As noted above, minK is a putative binding partner for telethonin and has been reported to localize to t-tubules at the Z-disc regions of cardiac and skeletal muscle (26). Nevertheless, as already discussed, minK expression is restricted to the conduction system (32), and its interaction with telethonin appears to be inhibited by phosphomimetic substitution of the Ser-157 residue of telethonin (26), making minK an unlikely t-tubular binding partner for the phosphorylated telethonin C terminus. Evidence suggests that junctophilin 2 (JP-2), a protein of the junctophilin family that is important for the formation of t-tubule/sarcoplasmic reticulum junctions in cardiomyocytes (36), may also regulate t-tubule organization (30). Consistent with this, reduced JP-2 protein expression in cardiomyocytes by lentiviral delivery of short hairpin RNA was found to disrupt t-tubule organization, as reflected by a significant reduction in the power of the dominant peak of t-tubule periodicity (30), in a manner analogous to our observations in ARVM following heterologous expression of non-phosphorylatable telethonin. It is tempting to speculate, therefore, that t-tubular JP-2 and titin-associated telethonin may both participate in a linker complex that anchors t-tubules to the Z-discs, thereby regulating t-tubule organization. Alternatively, telethonin may modulate the stability or function of such a complex in a phosphorylation-dependent manner. Regardless of the molecular mechanisms through which telethonin, and in particular its phosphorylated C-terminal tail, may maintain t-tubule organization, our findings suggest that this may be of functional importance in maintaining normal Ca^2+^*_i_* transients. In this context, the t-tubule and Ca^2+^*_i_* transient abnormalities that we have observed as a result of reduced telethonin phosphorylation (through the partial replacement of endogenous bis-phosphorylated telethonin with a non-phosphorylatable variant) are similar to those that have been reported in multiple cardiac pathologies, including heart failure (27, 31), and, recently, in ventricular myocytes from telethonin knockout mice (29). Ca^2+^*_i_* transient decay is regulated by a number of interacting factors, including the t-tubular Na^+^/Ca^2+^ exchanger and sarcoplasmic reticular Ca^2+^ ATPase activities. Although we have not investigated the contribution of these factors to the slowed Ca^2+^*_i_* transient decay that we have observed in cells expressing HA-S157A/S161A-telethonin, there is growing evidence that t-tubular disruption impairs Ca^2+^*_i_* extrusion via the Na^+^/Ca^2+^ exchanger (37).

Finally, are there potential causal roles for altered telethonin phosphorylation in cardiac pathophysiology? At present, it is not known whether non-phosphorylated telethonin or telethonin variants that lack the phosphorylated C-terminal tail may exist and have “poison peptide” effects that disrupt myocyte function in cardiac disease. In this context, it is interesting to note that several missense *TCAP* mutations associated with human hypertrophic and dilated cardiomyopathies introduce single residue substitutions within the C-terminal 36 amino acids of telethonin (9), which may interfere with the phosphorylation status of telethonin. Of these mutations, the potential impact of the R153H substitution (9) is of particular interest because of the proximity of Arg-153 to the phospho-acceptor residues of telethonin at Ser-157 and Ser-161. It is also interesting to note that *TCAP* mutations associated with limb-girdle muscular dystrophy type 2G introduce premature stop codons at the junction of exon 1 and intron 1 or in exon 2 (8). Although immunohistochemistry and immunoblot analysis of skeletal muscle samples from affected patients, some of whom also exhibited heart involvement, revealed the absence of telethonin protein, a polyclonal antibody raised against a C-terminal, 128-amino acid telethonin fragment was used (8). Thus, expression of truncated N-terminal telethonin fragments that lack the phosphorylated C-terminal tail cannot be excluded (particularly because *TCAP* has only two exons, with exon 1 coding for amino acids 1–37). In this regard, Pinotsis *et al.* (5) have shown that a truncated N-terminal telethonin fragment comprising amino acids 1–90 can be heterologously expressed in a stable manner (at least in neonatal rat cardiomyocytes) and that, like the full-length protein, it localizes to the sarcomeric Z-disc. The novel findings reported in this study form a strong basis for future work on the structural and functional roles of the C-terminal tail of telethonin and its phosphorylation-regulated interactions, particularly in the context of genetic cardiomyopathies associated with telethonin mutations.

## Footnotes

5 The abbreviations used are:

CaMK: Ca^2+^/calmodulin-dependent kinase
PKD: protein kinase D
ARVM: adult rat ventricular myocyte(s)
t-tubule: transverse tubule
CaMKII: Ca^2+^/calmodulin-dependent kinase II
PE: phenylephrine
λPPase: λ phosphatase.
